# Immunomodulation and Mechanical Characterization of Manuka Honey-Incorporated Near-Field Electrospun Bioresorbable Vascular Grafts

**DOI:** 10.3390/bioengineering12111270

**Published:** 2025-11-19

**Authors:** Alexandra E. Snyder, Evan N. Main, Gary L. Bowlin

**Affiliations:** Department of Biomedical Engineering, University of Memphis, Memphis, TN 38152, USA; esnyder2@memphis.edu (A.E.S.); enmain@memphis.edu (E.N.M.)

**Keywords:** near-field electrospinning, Manuka honey, neutrophils, polydioxanone, vascular graft

## Abstract

(1) Current synthetic small-diameter vascular grafts fail frequently due to anastomotic hyperplasia and thrombosis caused by mechanical mismatch and incomplete reendothelialization. Polydioxanone near-field electrospun (NFES) vascular templates feature programmable pore sizes to facilitate transmural ingrowth of endothelial cells and show promise in reducing mechanical mismatch, but their potential as drug delivery systems remains unexplored. It was hypothesized that Manuka honey incorporation in NFES templates could reduce neutrophil extracellular trap (NET) release but decrease mechanical strength. (2) Templates were fabricated using 90 mg/mL polydioxanone in 1,1,1,3,3,3-hexafluoro-2-propanol (HFP) and Manuka honey concentrations of 0%, 0.1%, 1%, and 10% *v*/*v*. Wall thickness (197–236 μm), mechanical properties, Manuka honey elution, and NET release were quantified. (3) The 0.1% and 1% templates best mimicked native vessel mechanics, outperforming the pure HFP template in tensile strength and burst pressure. The 10% templates exhibited significant mechanical strength reductions. Manuka honey elution exhibited a burst release within the first three hours, and all honey was eluted by day three. NET release was elevated in 10% and control groups but was not significantly different from 0.1% and 1%. (4) Overall, low concentrations of Manuka honey maintained mechanical compatibility, but elution must be optimized for immunomodulation, rejecting the initial hypothesis.

## 1. Introduction

Cardiovascular disease (CVD) is a global health crisis, responsible for approximately 1 in 3 deaths annually [1]. Atherosclerosis, the accumulation of fatty plaques along the arterial walls, is the most common cause of CVD. These plaques cause the arterial lumen to narrow, increasing the risk of limb amputation, heart attack, and stroke due to ischemia [2]. One current end-stage surgical treatment, aiming to restore blood flow to ischemic tissues, involves bypass grafting via either autologous or synthetic vascular grafts. Sources of autologous grafts, which are healthy vessels transplanted from other regions of the patient’s body, are limited due to widespread CVD and the need for multiple grafts [3,4,5]. Off-the-shelf synthetic vascular grafts, such as expanded polytetrafluoroethylene (ePTFE), perform well in applications with internal diameters larger than 6 mm, typically remaining patent for over 10 years [6,7]. However, small-diameter applications experience high rates of failure, with over one-third of implanted grafts failing within the first six months and more than half failing within two years [8,9,10]. Major contributors to graft failure in these small-diameter applications are compliance mismatch at the anastomosis and lack of reendothelialization which lead to anastomotic hyperplasia and thrombosis [11,12,13]. In extreme cases, graft failure can result in heart attack, stroke, and limb amputation, the same complications of atherosclerosis that bypass grafting is intended to prevent. Development of a bioresorbable vascular tissue template capable of mimicking properties of the native vasculature and minimizing inflammatory response while guiding fully functional in situ arterial regeneration poses a solution to these limitations.

Synthetic vascular grafts fail acutely due to thrombosis. The ideal antithrombotic surface for vascular grafts is the native endothelium, but reendothelialization is limited in current synthetic vascular grafts [14,15,16]. In animal models, reendothelialization of vascular grafts is achieved via transanastomotic ingrowth of endothelial cells, in which endothelial cells from the bypassed native artery migrate inwards bilaterally from the anastomoses [17]. However, humans do not achieve complete graft reendothelialization via this model, as endothelial cell migration is limited to 1–2 cm from the anastomosis over the course of several years with peripheral bypass grafts typically measuring 40–60 cm in length [18,19,20]. Instead, humans heal via transmural ingrowth of endothelial cells, in which endothelial cells from marginal tissue migrate to the luminal surface of the graft via capillaries derived from the exterior graft walls [21,22,23]. Standard ePTFE grafts do not possess pores large enough for transmural ingrowth to occur, preventing reendothelialization of small-diameter grafts. This lack of endothelial healing combined with the compliance mismatch of the graft and native artery cause a persistent injury response to the foreign material, increasing the risk of thrombosis and anastomotic hyperplasia [11,24].

The presence of large pores in a vascular graft may seem counterintuitive or unfeasible; however, synthetic grafts with high water permeability have been in use for decades [25,26]. Polyethylene terephthalate (PET) is another common synthetic vascular graft material used in both large- and small-diameter applications and has varying degrees of water permeability depending on the fabrication technique. When woven, PET grafts have low water permeability but, when knitted, are highly permeable to water [26]. To prevent hemorrhage and serum leakage, the highly water-permeable grafts are pre-clotted with patient blood, forming the provisional fibrin matrix [27]. This matrix prevents blood from leaking from the graft while still supporting the migration and infiltration of leukocytes, fibroblasts, and endothelial cells [28]. Several other techniques that do not require the use of patient blood have also been developed for cases that require the patient be on anticoagulants, including immersion in topical thrombin, sealing with fibrin glue, and impregnating with collagen [29,30]. However, these water-permeable PET grafts still exhibit a compliance mismatch with the native vasculature [31].

The key factors to developing the ideal vascular tissue template are pore sizes sufficient for ingrowth of endothelial cells, mechanical properties that mimic the native vasculature, and regulation of the immune response to the foreign material. Traditional electrospinning (TES) is a common technique used to fabricate templates for tissue engineering [32,33,34,35]. However, pore size and fiber diameter are intrinsically linked in this technique, so obtaining the 60–200 μm pore sizes necessary for angiogenesis requires a template fiber diameter impractical for vascular applications [36,37,38,39]. Near-field electrospinning (NFES) overcomes this limitation by reducing the airgap and pairing the precise relative motion of the charged spinneret and grounded collector mandrel to define pore sizes independent of fiber diameter [40,41,42]. Additionally, this precise relative motion allows for the fabrication of templates with precise fiber alignment that mimics the arterial extracellular matrix. Previous NFES work in vascular template fabrication demonstrated that, of the angles tested, a fiber alignment of 15° from the circumferential axis of the mandrel not only mimics the orientation of the vascular extracellular matrix but also closely mimics the mechanical properties of the saphenous vein (SV) and internal mammary artery (IMA), two vessels commonly used in autologous vascular grafting [43,44]. While these templates pose a solution to mechanical mismatch and lack of reendothelialization, they have yet to address potential for modulating immune response.

When a biomaterial is implanted, the initial immune response plays a significant role in whether the implant heals or fails. Neutrophils are phagocytic cells of the innate immune system and are a significant component of the wound healing process. Immediately after implantation, neutrophils swarm the wound site, upregulating genes that encode proteins involved in cell signaling for wound healing, including recruitment of macrophages and fibroblasts, as well as promotion of angiogenesis [45]. In fact, neutrophils are essential to angiogenesis as neutropenia results in complete inhibition of the revascularization of islets of Langerhans transplants in striated muscle, whereas complete revascularization is achieved in the presence of normal neutrophil counts [46]. Specifically, neutrophil extracellular traps (NETs), which condition the microenvironment of the wound, have been found to promote angiogenesis [47,48]. NETs are composed of modified chromatin coated with histones, proteases, and antibacterial proteins such as neutrophil elastase and myeloperoxidase (MPO). NETs are released in response to bacterial and inflammatory signals. While neutrophil response is essential to wound healing, dysregulated neutrophil activation and NET release can lead to chronic inflammation, further tissue damage, and formation of a fibrous capsule, inhibiting proper healing of the implanted biomaterial [49,50,51]. Excessive amounts of the dense, fibrous NET extrusions can collect on the surface of the implant, causing an NET-derived fibrosis. This impedes the migration and infiltration of other cells to the implant surface, delaying epithelialization in soft tissue implants, suggesting potential for a delay in reendothelialization of vascular grafts, increasing the risk of thrombosis [52]. Additionally, excessive excretion of MPO is associated with poor prognosis in CVDs [50,53]. Regulating neutrophil activation and preventing fibrous encapsulation of the implanted biomaterial are essential for the success of tissue-engineered vascular grafts requiring transmural angiogenesis.

In recent years, there has been an increase in studies investigating the potential of Manuka honey as a biomaterial additive due to its antibiotic and anti-inflammatory properties [54,55]. Manuka honey is derived from the nectar of the leptospermum scoparium shrub in New Zealand. Its methylglyoxal content is responsible for Manuka honey’s potent antibiotic quality, while its flavonoids and phenolic compounds provide its anti-inflammatory effects [54]. In a previous study, TES templates fabricated with Manuka honey demonstrated the ability to reduce NET release within a therapeutic window of 0.1–1%, whereas higher concentrations of Manuka honey induced cytotoxicity and increased NET release [56]. However, in a study solely testing whole Manuka honey’s ability to reduce NET release, in which Manuka honey was not incorporated into an electrospun template, larger concentrations of Manuka honey, up to 10%, still reduced NET release [57].

In this study, serial concentrations of Manuka honey were incorporated into NFES templates with a fiber alignment of 15° from the circumferential axis to study Manuka honey’s ability to regulate NET release in response to polydioxanone vascular templates in vitro. The templates were cultured with primary human neutrophils for 4 h, and their degree of NET release was quantified using an MPO enzyme-linked immunosorbent assay. Manuka honey templates were also mechanically characterized to determine the extent to which the addition of Manuka honey altered the mechanical properties of the vascular templates. It was hypothesized that the incorporation of Manuka honey into NFES vascular templates would reduce NET release within a therapeutic window but would cause a significant decrease in the mechanical properties of the templates.

## 2. Materials and Methods

### 2.1. Vascular Template Fabrication

A consumer-grade 3D printer (Prusa 12″ Basic Pegasus and Makerfarm, South Jordan, UT, USA) was adapted to create a custom NFES apparatus, as previously described [58]. The NFES printhead was a KDS Legato 130 single-syringe nanoliter infusion/withdrawal pump with a remote syringe pump head (KD Scientific Inc, Holliston, MA, USA), mounted on a custom fixture. The syringe pump held a 1 mL polypropylene syringe (Cat. No. 14955456, Fisher Scientific, Waltham, MA, USA) with a blunt, 23-gauge, 2-inch stainless steel Luer-lock needle (Cat. No. 75165A255, McMaster Carr, Elmhurst, IL, USA), powered by a DC voltage source (HV050REG (+), Information Unlimited, Amherst, NH, USA). The printhead translated along the *X*-axis, while a grounded 4 mm diameter stainless steel mandrel rotated about the *Y*-axis for fiber collection. The translation of the printhead was programmed using a custom G-code, communicated through the software Repetier-Host V.2.05 (Hot-World GmbH and Co. KG, Willich, Germany), while the *Y*-axis rotation was achieved using a stepper motor with an integrated driver and encoder (STM17S-1AE, Applied Motion Products, Watsonville, CA, USA) managed by the software Q Programmer V1.6.16 (Applied Motion Products, Watsonville, CA, USA).

Manuka honey solvent solutions were prepared by dissolving 0.2 mL of Manuka honey (ManukaGuard, MGO 400, Monterey, CA, USA) in 1.8 mL of 1,1,1,3,3,3-hexafluoro-2-propanol (HFP, Oakwood Products, Inc., Estill, SC, USA) in Class A clear glass threaded vials with PE Poly-Seal™ cone cap liners (Cat. No. 14955334, Fisher Scientific, Waltham, MA, USA) to create at 10% *v/v* Manuka honey solvent solution. The solvent solution was sonicated at room temperature for 4 h to ensure all visible honey particulates were dissolved. Serial dilutions were performed to make 0.1% and 1% *v/v* Manuka honey solvent solutions. Pure HFP was used as a control. Polydioxanone (DIOXOMAXX 100, inherent viscosity 1.70 dL/g, Bezwada Biomedical, LLC, Hillsborough, NJ, USA) was added to the solvent solutions at concentrations of 90 mg/mL. All templates were fabricated using a 1.7 mm airgap and a polymer flow rate of 18 μL/h with an applied voltage of 1.6 kV. The rotational velocity of the mandrel was 6.15 rev/s, and the translational velocity of the print head was 1242 mm/min, to achieve a resultant fiber angle of 15° with a resultant polymer jet velocity of 80 mm/s using calculations previously described [43]. For each vascular template, the syringe translated 30,000 times. After fabrication, the templates were wetted with 70% *v/v* isopropyl alcohol to ease removal from the mandrel, air-dried, and stored in a desiccator until further use.

Template morphology and fiber alignment were analyzed using scanning electron microscopy (SEM). Small cutouts of templates were sputter-coated with 5.0 nm of 60:40 gold–palladium in an argon atmosphere and visualized using SEM (Nova Nano 650 FEG, FEI Co., Hillsboro, OR, USA) with the field emission gun emitting +20 kV, a spot size of 1.5, and a 5 mm working distance. The fiber diameters and pore sizes were measured using the General Image Fiber Tool in ImageJ (National Institutes of Health, Public Domain, BSD-2, Bethesda, MD, USA) [59]. Wall thickness of every sample was measured before each mechanical test using a digital groove thickness gauge (Model 547-516S, Mitutoyo, Aroura, IL, USA). Fiber diameter, pore size, and wall thickness data for each template are reported as the mean and standard deviation in micrometers (μm).

### 2.2. Mechanical Characterization

Mechanical properties of the vascular templates were evaluated via tensile testing until failure. Longitudinal and circumferential elongation and suture retention were performed using a uniaxial testing frame (Model Q CONTROLLER, Test Resources, Shakopee, MN, USA), equipped with a 25 lbf load cell (Model SM-25-294, Test Resources, Shakopee, MN, USA). Burst pressure testing was performed using a custom apparatus equipped with a syringe pump (Model 300, New Era Pump Systems Inc., NY, USA) and pressure transducer (Model G2 Pressure Transducer, Ashcroft, Stratford, CT, USA). All vascular templates were fully submerged in Hank’s balanced salt solution (HBSS, ref. no. 14175-095, Gibco, Waltham, MA, USA) and incubated at 37 °C for 90 min to hydrate and equilibrate to physiological temperature. Mechanical properties were evaluated for templates of each concentration of Manuka honey and compared to literature findings of the mechanical properties of the IMA, SV, and standard-wall, 4 mm Gore-Tex^®^ vascular grafts (Cat No. V04070L, W.L. Gore & Associates, Inc., Flagstaff, AZ, USA), which is a standard off-the-shelf ePTFE graft [60,61,62,63,64]. All testing procedures were lab-validated, conforming to standards set by the American National Standards Institute and the Association for the Advancement of Medical Instrumentation entitled “Cardiovascular Implants-Vascular Graft Prostheses” [65]. The data are reported as the mean and standard deviation of each template group per mechanical test, then plotted as box plots with the individual sample results overlayed.

### 2.3. Longitudinal Uniaxial Elongation

Vascular templates were cut perpendicular to the long axis into 20 mm cylindrical segments (*n* = 6). Each end was secured in a knurled grip vise with an initial separation of 8 mm. The grips were separated at a rate of 50 mm/min until failure with a sampling rate of 50 samples per second. The data is recorded in Newtons (N), and the ultimate tensile stress is reported in megapascals (MPa) based on the initial cross-sectional area. Total percent elongation is reported as a percentage based on the initial grip separation.

### 2.4. Circumferential Uniaxial Elongation

Vascular templates were cut perpendicular to the long axis into 4 mm cylindrical segments (*n* = 6). Two 1.2 mm diameter pins were slid through the lumen of the segment and secured to a custom apparatus in the knurled vise grips of the testing frame with an initial pin separation of 4 mm. The grips were separated at 50 mm/min until failure at a sampling rate of 50 samples per second. The data is recorded in Newtons (N), and the ultimate tensile stress is reported in megapascals (MPa) based on the initial cross-sectional area. Total percent elongation is reported as a percentage based on the initial pin separation.

### 2.5. Suture Retention

Vascular templates were cut into 20 mm cylindrical segments, perpendicular to the long axis, and a single wall was threaded with a 5-0 Redilon™ Monofilament Polymide Nylon Non-Absorbable Surgical Suture U.S.P. (MYCO Medical, Cary, NC, USA) to form a half loop 2 mm below the template cut line (*n* = 6). The bottom of the template was fixed in the lower knurled grip vise, and the suture was fixed in the opposing knurled grip vise with an initial grip separation of 13 mm. The grips were separated at a rate of 150 mm/min until failure at a sampling rate of 250 samples per second. The data is recorded and reported in grams-force (gf).

### 2.6. Burst Pressure

Vascular templates were cut into 40 mm segments and pulled over a 160Q latex balloon segment (Qualatex, Wichita, KS, USA) (*n* = 6). The sample was then fixed to the barbed hose fittings of the custom burst pressure apparatus using 2-0 Black Braided Silk non-resorbable sutures (SP118, Surgical Specialties Corp., Westword, MA, USA). Water pressure inside the balloon channel was increased by 15 mm of mercury per second (mmHg/s) until graft rupture. The maximum pressure data was measured and is reported in mmHg.

### 2.7. Template Sterilization

Templates were sterilized before use in the NET release and Manuka honey elution studies. Circular punches were taken from templates of all Manuka honey concentrations using an 8 mm diameter biopsy punch (cat. no. P825, Acuderm Inc., Ft. Lauderdale, FL, USA) and stored in a desiccator until use. In a sterile, laminar hood, the templates were placed in a 96-well plate and sterilized for at least 10 min on each side with ultraviolet light at a wavelength of 365 nm using an 8 W lamp (cat. no. EN280L, Spectroline, Westbury, NY, USA) at a working distance of 9.5 cm [66].

### 2.8. Manuka Honey Elution

Manuka honey elution from NFES vascular templates was evaluated via glucose assay as previously described [67]. Templates were then incubated in 200 μL of 1 x phosphate-buffered saline (PBS) (PBS, ref. no. 10010-023, Gibco, Waltham, MA, USA) for 1, 3, 6, and 24 h, and 2, 3, 7, 14, and 21 days at 37 °C and 5% CO_2_. At each time point, the supernatant was collected from each sample in 1.5 mL microcentrifuge tubes and stored at −20 °C until analysis. The sample wells were replenished with 200 μL of PBS and continued incubation until the next time point. Collected samples were thawed for use in a colorimetric glucose assay (Glucose Assay Kit, cat. No. MAK476, Sigma Aldrich, St. Louis, MO, USA), and the absorbance was read on a SpectraMax i3x Multi-Mode Microplate Reader (Molecular Devices, San Jose, CA, USA) at 570 nm. The glucose concentration was determined from a glucose standard curve ranging from 0 to 300 μM of glucose. Additionally, a standard dilution of Manuka honey ranging from 0% to 10% was assayed to relate glucose elution to Manuka honey elution. Data are reported as mean Manuka honey concentration in mg/mL per template group at each time point (*n* = 4).

### 2.9. NET Release

Four separate experiments were conducted using fresh peripheral blood neutrophils isolated from four individual healthy donors of randomized race and sex, without a history of endocrine, autoimmune, cardiovascular, or inflammatory diseases. The experiments followed protocols approved by the University of Memphis Institutional Review Board (IRB ID: #PRO-FY2020-230) which included informed consent. Donors were instructed to avoid alcohol consumption and non-steroidal anti-inflammatory drug use within 48 h and not to eat or consume caffeine within 12 h of blood donation [57].

Neutrophils were then isolated from the collected peripheral blood via density separation using Isolymph^®^ (CTL, Deer Park, NY, USA, density 1.077 ± 0.001g/mL, ref. no. 759050) [57,68,69]. After isolation, neutrophils were resuspended in HBSS without calcium and magnesium with 10 mM N-2-hydroxyethylpiperazine-N-2-ethane sulfonic acid (HEPES, ref. no. 25-060-CI, Corning, Corning, NY, USA) and 0.2% autologous serum (denoted as HBSS+ hereafter) at a concentration of 1 million neutrophils/mL. The disinfected biomaterials (*n* = 3) were placed in a 96-well plate, and 40 μL of HBSS+ was added to each well to hydrate the templates. Negative control tissue culture plastic (TCP) wells (*n* = 3) received 40 μL of HBSS+, and positive control TCP wells (*n* = 3) received 30 μL of HBSS+ prior to cell seeding to ensure all wells had a final volume of 150 μL. Then, 100 μL of the resuspended cell solution containing 100,000 neutrophils was added to each well followed by 10 μL of heparin (cat. no. H3393, Sigma–Aldrich, St. Louis, MO, USA) at a final concentration of 10 U/mL heparin to dissociate NET-associated MPO [56]. The negative control TCP wells received no added stimulant, and the positive control TCP wells were stimulated with 10 μL of 100 nM phorbol 12-myristate 13-acetate (PMA, cat. no. P8139, Sigma–Aldrich, St. Louis, MO, USA) in HBSS+. The neutrophils were cultured at 37 °C and 5% CO_2_ for 4 h. Following incubation, the samples were placed on ice for 10 min to inhibit further neutrophil stimulation during supernatant collection. Supernatants were collected in 1.5 mL microcentrifuge tubes and centrifuged for 5 min at 500× *g* at room temperature in a Sorvall Legend XTR Centrifuge (Thermo Scientific, Waltham, MA, USA), then transferred to fresh microcentrifuge tubes, and stored at −20 °C until analysis [70]. NET release was quantified using a human MPO enzyme-linked immunosorbent assay (Cat. No. BMS2038INST, ThermoFisher Scientific, Waltham, MA, USA) following methods as previously described [70]. Due to high variability in neutrophil inflammatory responses per donor, all data were normalized to the positive controls per donor and are expressed as mean percent NETs per template.

### 2.10. Statistical Methods

All statistical analyses were performed in MATLAB R2025a (MathWorks Inc., Natick, MA, USA). A Shapiro–Wilk test was performed to test for normality. If the data sets were normal, one-way ANOVA and post hoc Tukey–Kramer tests were performed at a significance of *p* < 0.05 to determine any significant differences in the performances of the various Manuka honey concentrations. If the data was not normal, Kruskal–Wallis non-parametric ANOVA and Dunn post hoc tests were performed. Data are reported as the mean ± standard deviation.

## 3. Results

### 3.1. Template Morphology

**Figure 1:**
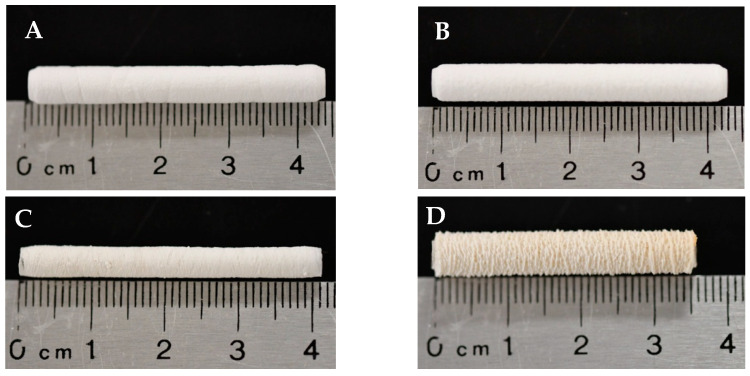
Macro-images of the (**A**) pure HFP and (**B**) 0.1%, (**C**), 1%, and (**D**) 10% Manuka honey NFES vascular templates.

**Figure 2:**
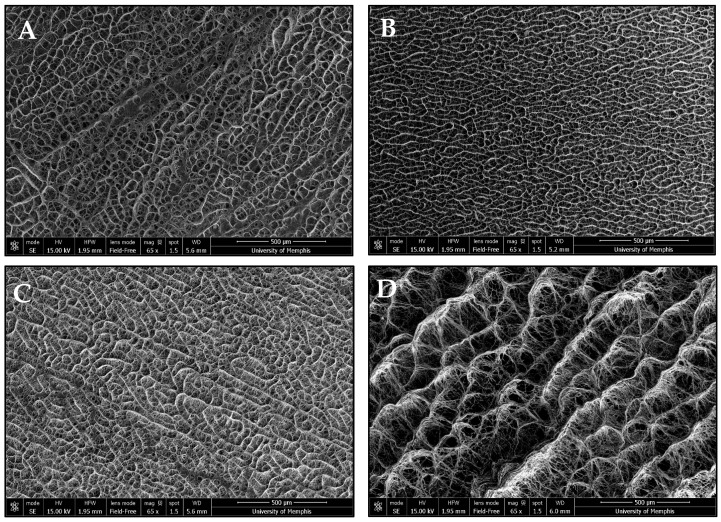
Representative SEM images of the outer surface of the (**A**) pure HFP template, (**B**) 0.1% Manuka honey template, (**C**) 1% Manuka honey template, and (**D**) 10% Manuka honey template. All images were taken at 65× magnification (scalebar = 500 μm).

**Figure 3:**
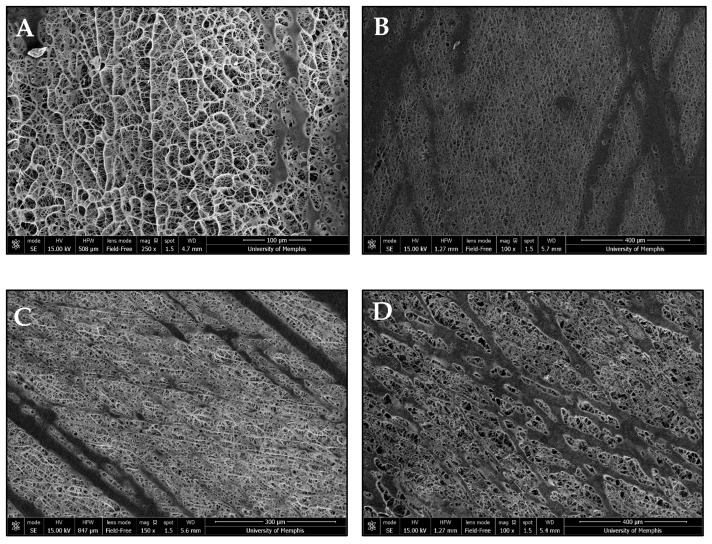
Representative SEM images of the mandrel facing surface of the (**A**) pure HFP template (scalebar = 100 μm), (**B**) 0.1% Manuka honey template (scalebar = 400 μm), (**C**) 1% Manuka honey template (scalebar = 300 μm), and (**D**) 10% Manuka honey template (scalebar = 400 μm).

NFES produced continuous, tubular vascular templates. The incorporation of Manuka honey caused macroscopic changes in template morphology (Figure 1). Microscopic template morphology was analyzed via SEM (Table 1). Fiber diameter and pore size were measured using micrographs of the outer surface of the templates (Figure 2). However, the programmed fiber alignment is most prominent on the inner surface of the templates (Figure 3). The fibers of the pure HFP, 0.1%, and 1% templates were densely packed, whereas the fibers of the 10% template were loosely packed with larger pores. There was no significant difference in any of the template fiber diameters, but the 10% template had significantly larger pores than all other templates. Additionally, the 0.1% template had significantly smaller pores than the 1% template. Template pore size increased with increased Manuka honey concentration. However, all templates possessed sufficient pore sizes to facilitate angiogenesis. There were no significant differences in wall thickness between any of the templates. Additionally, the 10% template was flaccid in nature, deflecting under its own weight when fixed at one end, while the other templates were stiffer, maintaining their shape when fixed at one end, but were still flexible.

### 3.2. Mechanical Characterization

**Figure 4:**
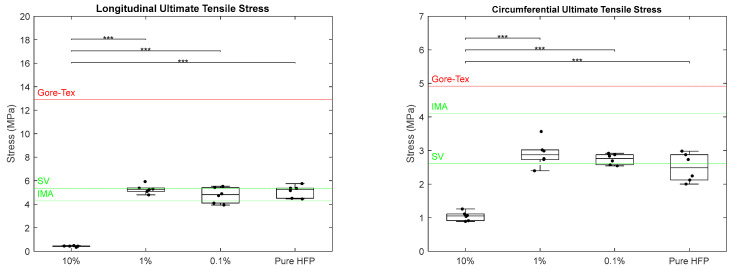
Ultimate tensile stress of Manuka honey vascular grafts along longitudinal axis (**left**) and circumferential axis (**right**). The red line indicates literature values for Gore-Tex^®^, the green line indicates literature values for SV and IMA, and the triple asterisk located at the top of the graphs indicates *p* < 0.001. Raw data available in Supplementary Spreadsheet S2.

Ultimate tensile stress was measured for each template in both the longitudinal and circumferential axes (Figure 4). In the longitudinal axis, there was a significant difference in the ultimate tensile stresses (UTSs) between the 10% template compared to every other template concentration. The 10% template had a UTS of 0.42 ± 0.05 MPa, while the 1%, 0.1%, and pure HFP templates had UTS values of 5.29 ± 0.37, 4.77 ± 0.66, and 5.10 ± 0.52 MPa, respectively. When compared to the native vascular values, the 10% graft did not meet either of the target values, whereas 1%, 0.1%, and pure HFP were all in between the IMA value of 4.3 MPa and the SV value of 5.34 ± 0.60 MPa [42,43]. None of the templates met the Gore-Tex^®^ value of 12.9 MPa [39]. Similarly, in the circumferential axis, there was a significant difference in the UTS values between the 10% Manuka honey template and all other templates with a UTS of 1.05 ± 0.14 MPa. The 1% and 0.1% templates were the only templates to surpass the SV value of 2.61 ± 0.67 MPa with UTS values of 2.91 ± 0.39 and 2.74 ± 0.16 MPa, respectively. The pure HFP templates were slightly below the SV target value with a UTS of 2.49 ± 0.42 MPa [43]. None of the templates met the IMA target value of 4.1 MPa or the Gore-Tex^®^ value of 4.9 MPa [39,42]. The standard deviation and/or standard error could not be found in the literature for the IMA and Gore-Tex^®^ values.

**Figure 5:**
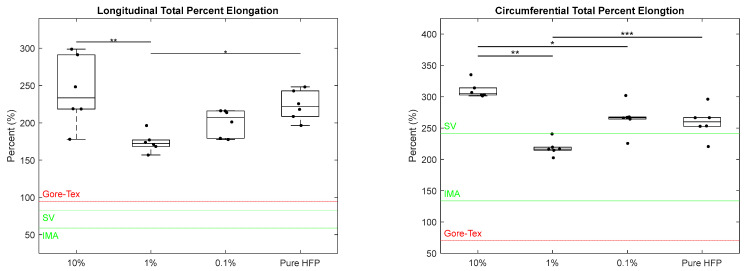
Total percent elongation of Manuka honey vascular grafts along longitudinal axis (**left**) and circumferential axis (**right**). The red line indicates literature values for Gore-Tex^®^ and the green line indicates literature values for SV and IMA. Single asterisks located at the top of the graphs indicate *p* < 0.05, double asterisks indicate *p* < 0.01, and triple asterisks indicate *p* < 0.001. Raw data available in Supplementary Spreadsheet S2.

The total percent elongation was calculated for each template in both the longitudinal and circumferential axes (Figure 5). In the longitudinal axis, the 10% template experienced the largest increase in length under mechanical load, with a total percent elongation of 242 ± 46.7%, followed by the pure HFP template with a total percent elongation of 223 ± 19.9%. Both the 10% and pure HFP templates had significantly higher total percent elongations than the 1% template which had a total percent elongation of 173 ± 12.9%. The 0.1% template was not significantly different from any of the other templates with a total percent elongation of 200 ± 18.1%. All templates surpassed both the IMA and SV target values of 59% and 83%, respectively, and the Gore-Tex^®^ value of 95.0% [39,42]. Similarly, in the circumferential axis, the 10% template experienced the largest increase in length under mechanical load with a total percent elongation of 310 ± 12.9%, which was significantly greater than those of the 1% and 0.1% templates that had total percent elongations of 218 ± 12.4% and 265 ± 24.3%, respectively. The pure HFP template had a significantly higher total percent elongation than the 1% template, measuring 259 ± 24.7%. The 1% template was the only template to not meet the SV target value of 242% [42]. However, all templates surpassed the IMA target value of 134% and the Gore-Tex^®^ value of 70.5% [39,42].

**Figure 6:**
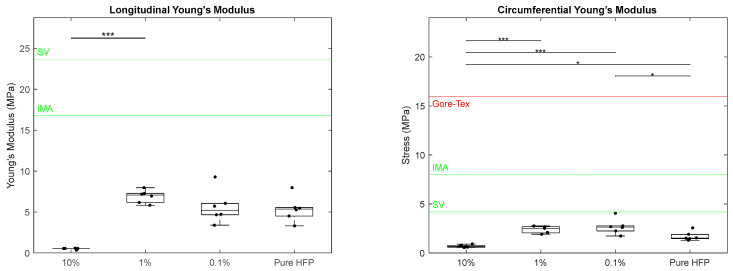
Young’s modulus of Manuka honey vascular grafts in the longitudinal axis (**left**) and circumferential axis (**right**). The red line indicates literature values for Gore-Tex^®^, the green line indicates literature values for SV and IMA, single asterisk located at the top of the graphs indicates *p* < 0.05, and the triple asterisk indicates *p* < 0.001. Raw data available in Supplementary Spreadsheet S2.

Young’s modulus was calculated for each template in both the longitudinal and circumferential axes (Figure 6). In the longitudinal axis, the Young’s modulus of the 10% template was significantly lower than that of the 1% template, measuring at 0.53 ± 0.09 MPa and 6.91± 0.78 MPa, respectively. The 0.1% and pure HFP templates had similar Young’s modulus values of 5.64 ± 2.0 MPa and 5.35 ± 1.53 MPa, respectively. None of the templates met the IMA and SV target values of 16.8 MPa and 23.7 MPa, respectively [42]. No literature value for Gore-Tex^®^ could be found for longitudinal Young’s modulus. In the circumferential Young’s modulus, the 10% template had a significantly lower value than those of all other templates with a modulus of 0.69 ± 0.17 MPa. The pure HFP template had a modulus of 1.71 ± 0.46 MPa, which was significantly lower than the 0.1% template’s modulus of 2.68 ± 0.77 MPa. The 1% template had a Young’s modulus of 2.39 ± 0.37 MPa. None of the templates met the SV target value of 4.2 MPa or the IMA target value of 8 MPa [42]. An error could not be found for the SV and IMA circumferential Young’s modulus values.

**Figure 7:**
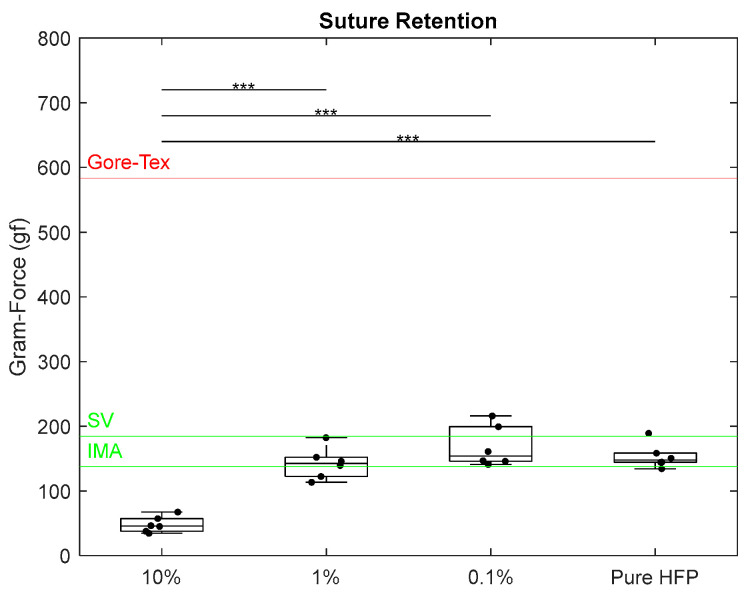
Suture retention of Manuka honey vascular grafts. The red line indicates literature values for Gore-Tex^®^, the green line indicates literature values for SV and IMA, and the triple asterisk located at the top of the graphs indicates *p* < 0.001. Raw data available in Supplementary Spreadsheet S2.

Suture retention testing measured the force required for template wall failure when a half loop suture is pulled from a single wall of the templates. The retention force of the 10% template was measured at 48.0 ± 12.3 gf, which was significantly lower than those of all other templates (Figure 7). All other templates exhibited retention forces between the IMA value of 138 gf and the SV value of 185 gf [40,41]. The 1% template fell closest to the IMA value with a retention force of 142 ± 24.3 gf. The 0.1% and pure HFP templates were slightly closer to the SV value with retention forces of 168 ± 31.5 gf and 153 ± 19.1 gf, respectively. None of the templates came close to reaching the Gore-Tex^®^ literature value of 584 gf [39].

**Figure 8:**
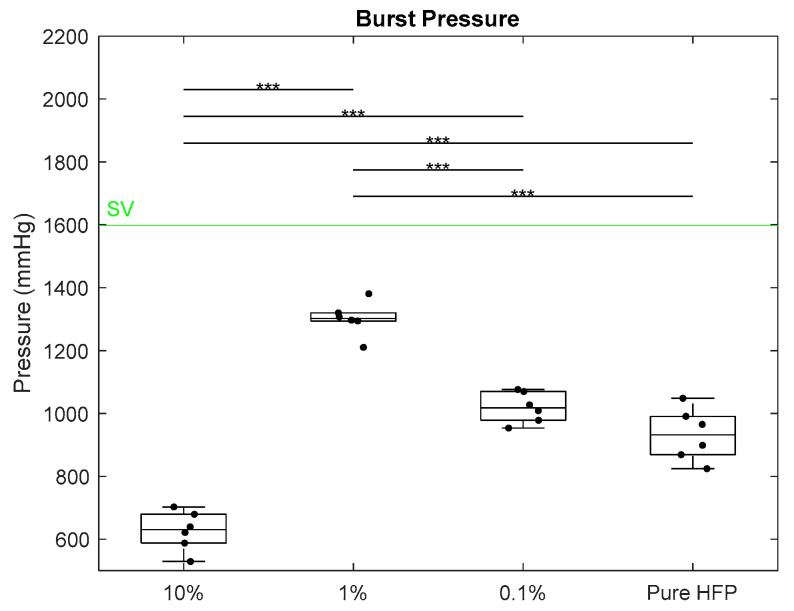
Burst pressure of Manuka honey vascular grafts. The green line indicates literature values for SV, and the triple asterisk located at the top of the graphs indicates *p* < 0.001. IMA and Gore-Tex^®^ values were omitted for visual clarity. Raw data available in Supplementary Spreadsheet S2.

Burst pressure testing measured the maximum pressure the templates sustained before wall rupture. None of the templates met the SV target value of 1599 ± 892 mmHg or the IMA target values of 3196 ± 1264 mmHg (Figure 8) [42]. Additionally, none of the templates reached the Gore-Tex^®^ burst pressure, which is assumed to be greater than 1900 mmHg [39]. The 10% template had a burst pressure of 627 ± 62.8 mmHg, which was significantly lower than those of all other templates. The 1% template had a significantly higher burst pressure than those of all other templates, measuring at 1302 ± 54.8 mmHg. The 0.1% and pure HFP templates had burst pressures of 1019 ± 48.9 mmHg and 993 ± 83.3 mmHg, respectively. While none of the templates met any of the mean native vasculature values, the mean burst pressures of the 1%, 0.1%, and pure HFP templates all fell within the lower error of the SV.

### 3.3. Manuka Honey Elution

**Figure 9:**
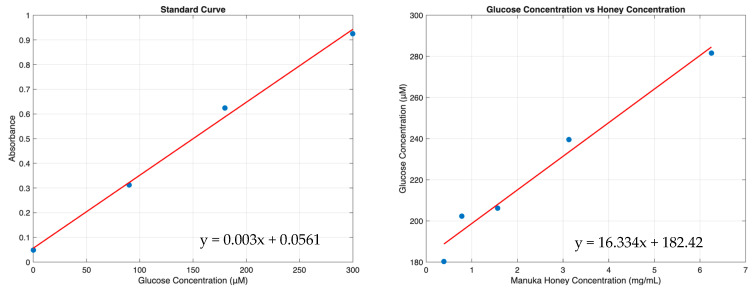
Curves demonstrating correlations between absorbance and glucose concentration, and the resulting glucose concentration vs. Manuka honey concentration curve. Raw data available in Supplementary Spreadsheet S3.

**Figure 10:**
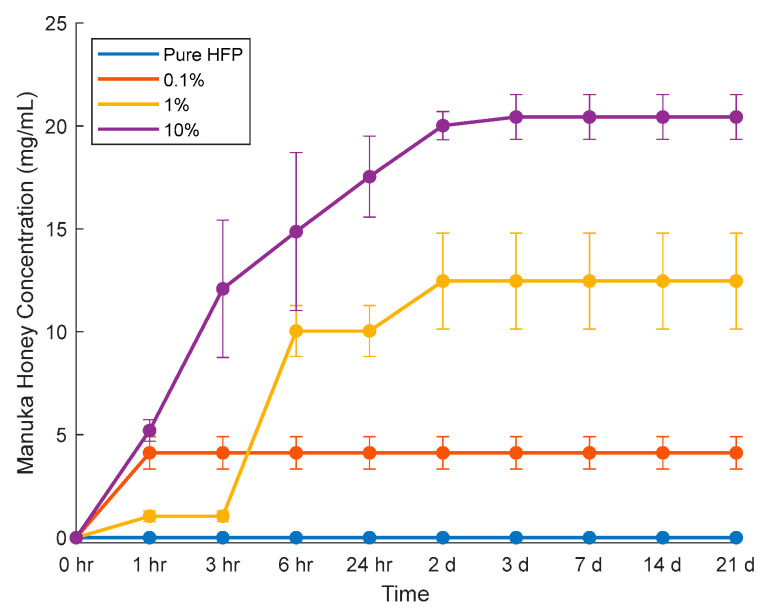
Manuka honey elution from vascular templates incubated in PBS. Raw data available in Supplementary Spreadsheet S3.

The elution study was performed by incubating templates in PBS. Glucose concentrations were measured via assay and converted to Manuka honey concentrations using a series of standard curves (Figure 9). The elution at each time point was summed to determine cumulative Manuka honey elution over time (Figure 10). The 10% template demonstrated a burst release resulting in a concentration of 12.1 ± 3.3 mg/mL of Manuka honey within the first three hours and then experienced a linear increase in Manuka honey released until day three, when the release plateaued at 20.4 ± 1.1 mg/mL. The 0.1% template had only a burst release of 4.11 ± 0.78 mg/mL within the first hour and did not continue to any more Manuka honey. The 1% template had the greatest Manuka honey release between hour three and hour six, releasing nearly 9 mg/mL of Manuka honey between these time points, and continued to release Manuka honey until day two.

### 3.4. NET Release

**Figure 11:**
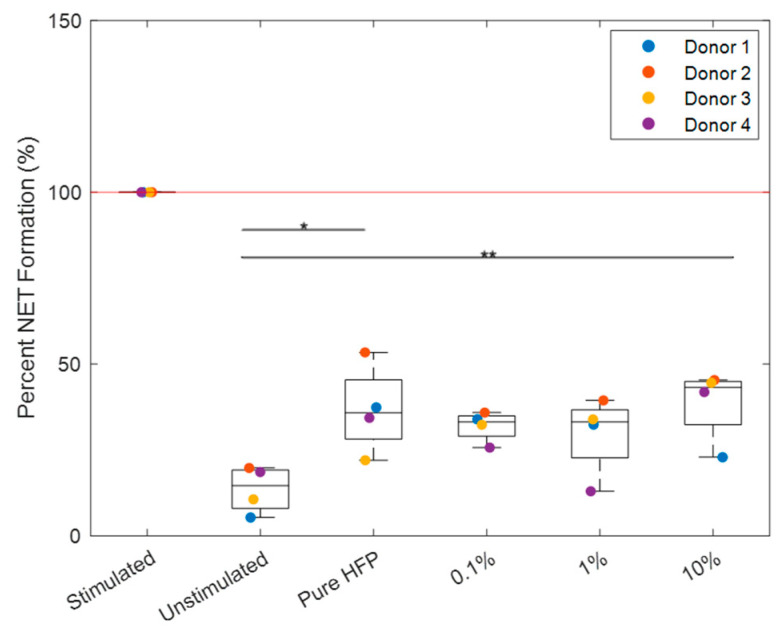
Percent NET release per Manuka honey template. The red line indicates the positive control. Single asterisks located at the top of the graphs indicate *p* < 0.05 and double asterisks indicate *p* < 0.01. Indicators for significant difference from the positive control were omitted for visual clarity. Raw data available in Supplementary Spreadsheet S4.

**Figure 12:**
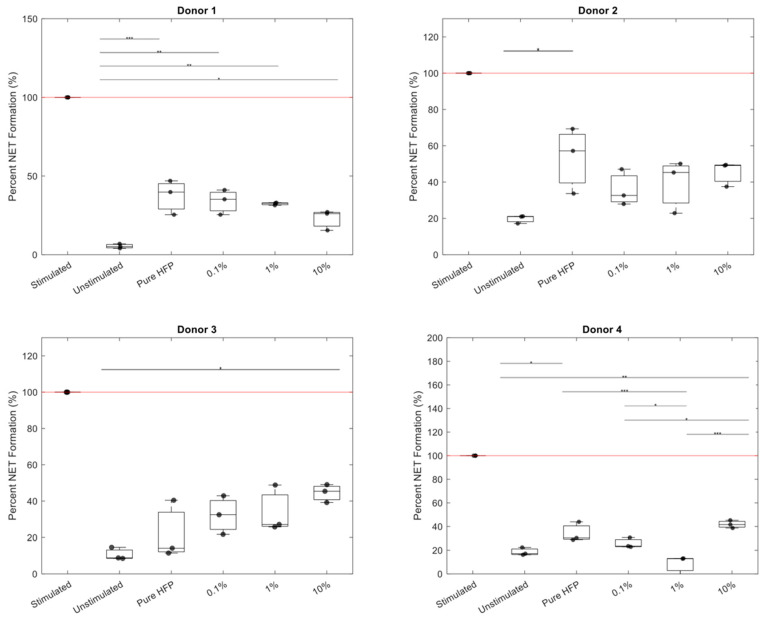
Percent NET release per Manuka honey template per donor. The red line indicates the positive control. Single asterisks located at the top of the graphs indicate *p* < 0.05, double asterisks indicate *p* < 0.01, and triple asterisks indicate *p* < 0.001. Indicators for significant difference from the positive control were omitted for visual clarity. Raw data available in Supplementary Spreadsheet S4.

NET release for each donor was normalized to their stimulated positive control (Figure 11). All templates exhibited a significantly lower NET release than the positive control with *p* < 0.001. The 0.1% and 1% templates were the only templates that did not exhibit a significantly higher NET release than the unstimulated control. While the pure HFP and 10% templates were significantly higher in value than the unstimulated control, they were not significantly different from the 0.1% and 1% templates. Additionally, the 0.1% and 1% templates were not significantly different in value than the unstimulated control. The 0.1% template reduced NET release in three out of the four donors, in which donor 3 had an increased NET release at each concentration of Manuka honey. There are no other discernible patterns in NET reduction, with each donor responding differently to the increases in Manuka honey concentration (Figure 12).

## 4. Discussion

The current small-diameter ePTFE grafts are not porous enough to facilitate the transmural ingrowth of capillaries necessary for complete reendothelialization and possess mechanical properties significantly different to that of the native vasculature, impeding blood flow and increasing the risk of thrombosis and graft occlusion. The development of a bioresorbable small-diameter off-the-shelf vascular graft that closely mimics the mechanical properties of the native vasculature and is porous enough to enable arterial regeneration is imperative for the improvement in long-term success rates of vascular grafts in small-diameter applications. NFES grafts have previously demonstrated promise in reducing mechanical mismatch by mimicking the arterial extracellular matrix and possessing programmable pore sizes to facilitate transmural ingrowth of endothelial cells. However, these grafts still need to address the potential for dysregulated immune response leading to dysregulated inflammation and a neutrophil NET-derived fibrosis [50].

To regulate immune response and prevent NET-derived fibrosis, bioactive agents can be incorporated into vascular templates to modulate acute inflammation. Manuka honey presents a potential solution to regulating immune response, acting as both a potent antibiotic and anti-inflammatory drug. Previously, Manuka honey has demonstrated the ability to reduce NET release in response to both whole Manuka honey solutions and TES templates. However, Minden-Birkenmaier et al.’s study demonstrated a reduction in NET release in response to TES templates within a therapeutic window, while Main et al.’s whole Manuka solutions demonstrated decreased NET release at all therapeutic doses [56,57]. This study aimed to incorporate Manuka honey into NFES vascular templates to characterize its effect on mechanical strength and NET release. It was hypothesized that incorporation of Manuka honey into these templates would reduce NET release but also significantly decrease mechanical strength.

The 0.1% and 1% templates exceeded expectations in mechanical characterization, achieving at least one native vascular target value in UTS in both axes, total percent elongation in both axes, and in suture retention. The 1% template also outperformed the pure HFP template in several mechanical tests, exhibiting a slightly higher UTS in both axes and Young’s modulus in both axes, as well as exhibiting a significantly higher burst pressure. The increased porosity from the addition of Manuka honey resulted in a decreased fiber density per unit area which theoretically could affect mechanical strength. However, the increase in pore size was not directly proportional to the decrease in burst strength. Specifically, the 1% template had a significantly higher burst strength than the 0.1% template, even though the 1% template had larger pores. The 1% template did have a thicker wall than the 0.1% template, which is more likely the cause of the difference in burst strength [71,72]. Future experimentation should include wall thickness and burst pressure correlation and optimization. Nevertheless, these findings contradict the hypothesis that the addition of Manuka honey would reduce mechanical strength. While the 1% template did have the lowest total percent elongation in the longitudinal axis compared to all other templates, it most closely mimicked the low longitudinal elasticity of the native vasculature. The 10% templates performed as expected, exhibiting significantly lower mechanical strengths than all other templates in 6 of the 8 reported tests. In the other two tests, with the total percent elongation in both axes, the 10% template exhibited higher percent elongations than all other templates but had the greatest difference from the native vasculature values. These results suggest that the addition of Manuka honey to vascular grafts in low concentrations does not impair, and may even enhance, mechanical performance, but high concentrations significantly decrease the mechanical strength of vascular templates.

Since Manuka honey is approximately 25% glucose, and glucose is a stable carbohydrate unlikely to react with or degrade in polar, nonnucleophilic solvents like HFP, it is assumed that measuring the glucose concentration of the releasate from the templates can be directly correlated with the concentration of Manuka honey released from the templates [67,73,74,75]. The 10% template had the highest concentration of released Manuka honey compared to all other templates within the first three hours and therefore had the greatest amount of Manuka honey present during the neutrophil studies. The 0.1% template had the second highest Manuka honey concentration within the first three hours. However, because Manuka honey elution was not measured at hour 4 and the 1% template surpassed the 0.1% template’s total elution between hour 3 and hour 6, it is unknown whether the 1% or 0.1% template had the highest Manuka honey concentration by the time of supernatant collection. The rapid elution of Manuka honey from the 0.1% template within the first hour is ideal for targeting the initial influx of neutrophils at the beginning of the inflammation stage of the wound healing process. The 1% and 10% templates eluted all their Manuka honey load by days two and three, respectively. This timeline corresponds with the overall duration of neutrophil activity at the wound site, as neutrophil activity begins to decline 2–3 days post injury [76].

NET release was quantified by measuring MPO in collected supernatants because NET release is dependent on MPO and MPO quantification, which has been demonstrated to be equally as effective as fluorescent imaging analysis [70,77]. In the NET release results, both the pure HFP and 10% templates exhibited significantly a higher NET release than the unstimulated control. However, no significant differences were observed between the two templates, indicating that the 10% template performed comparably to a template with no drug load. While the 0.1% and 1% templates did not exhibit a significant decrease in NET release compared to the pure HFP and 10% templates, they also did not exhibit a significantly higher NET release than the unstimulated control. Since the pure HFP template did exhibit a significant increase in NET release compared to the unstimulated control, the lack of significant increase between the unstimulated control and the 0.1% and 1% templates suggests that the addition of Manuka honey into the templates did prevent an increase in NET release. However, there was high donor-to-donor variability, as all four donor’s neutrophils behaved differently when exposed to the Manuka honey-incorporated templates. Donors 2 and 4 exhibited an increased NET release in the 10% template as described by Minden-Birkenmaier et al., while Donor 1 exhibited a decreased NET release at all concentrations of Manuka honey as described by Main et al. [56,57]. Overall, these results indicate that low concentrations of Manuka honey may mitigate excessive NET release, though the extent of modulation will ultimately vary on a case-by-case basis.

This study was designed to minimize variability in donor immune response as much as possible, and the results from each donor were normalized to their respective stimulated control to mitigate baseline inflammatory differences detectable in the data. However, variability is inevitable. Although donors were instructed to follow specific criteria prior to donation, they may not have been truthful about their habits at the time of donation. Additionally, donors could have experienced heightened inflammatory responses due to asymptomatic or unreported infections. Despite donor variability, overall NET response patterns revealed meaningful differences between Manuka honey concentrations.

An increase in NET release in response to the 10% templates, as observed in Minden-Birkenmaier et al.’s study, was expected as both studies were conducted with polydioxanone electrospun templates. However, NET release in response to the 10% template was attenuated in the NFES templates compared to Minden-Birkenmaier et al.’s TES templates, as the TES 10% templates demonstrated significantly more NET release compared to the TES control template, whereas the NFES 10% template did not have a significantly different NET release compared to the NFES pure HFP template. This finding is consistent with a study by King et al. that demonstrated attenuation of NET release in response to NFES templates compared to TES templates, hypothesizing that because the pores in NFES templates are larger than neutrophil bodies, the neutrophil does not detect the material and therefore does not react [39]. Neutrophils are 12–15 μm in diameter, while the pores of the NFES templates in this study ranged from 74.5 to 128 μm in diameter, suggesting that the large pore sizes may have attenuated NET release independent of the addition of Manuka honey [78].

Because the purpose of this study was solely to characterize Manuka honey’s effect on template mechanical properties and template-induced neutrophil activation, all electrospinning parameters were kept the same, with the only variable throughout this study being the volume of Manuka honey in the solvent solutions. The 10% template was the most challenging to fabricate, so all electrospinning parameters were standardized based on the optimal parameters for the 10% template. While the 1% and 0.1% templates significantly outperformed the 10% template in mechanical strength, fabrication parameters of the 1% and 0.1% templates could be optimized to more accurately mimic the mechanical properties of the native vasculature. Due to this lack of optimization, template fiber architecture may have affected NET release. Neutrophils are less reactive to large-diameter fibers (1.0–2.00 μm) than small-diameter fibers (0.25–0.35 μm) even in the presence of certain anti-inflammatory drugs [37,56,66,68]. A study by Fetz et al. investigated the effect of chloroquine elution from TES templates on NET release and found that large fiber templates loaded with chloroquine did not significantly differ from the large fiber control, whereas the corresponding small fiber templates did exhibit a significant reduction in NET release even though both the small and large fiber templates eluted equivalent levels of chloroquine at each time point [66]. The NFES templates in this study possessed large-diameter fibers, meaning they were favorable to neutrophils prior to the addition of Manuka honey. This favorability of the template fiber diameters in addition to the attenuation of NET release on NFES templates compared to TES templates with similar fiber diameters suggest that the architecture of the NFES templates alone reduces NET response and the addition of Manuka honey may not result in a significant reduction in immune response [39]. Further, large-diameter fibers typically do not elute the loaded drug as readily as small-diameter fibers, so the amount of Manuka honey eluted from the templates may be insufficient to cause a significant decrease in template-induced NET release [39,56]. Nevertheless, with the current template architecture, the 0.1% and 1% Manuka honey templates were still capable of preventing a significant increase in NET release compared to the unstimulated control, while the unloaded template did exhibit a significant increase in NET release.

Previous studies have demonstrated that, in the presence of high glucose concentrations, cells metabolize the glucose into methylglyoxal, the potent antibiotic component of Manuka honey, which in high doses can induce an increase in neutrophil intracellular reactive oxygen species, mitochondrial damage and apoptosis in liver cells, and microvascular damage in the blood–brain barrier [79,80]. The high glucose concentration in the 10% template could have resulted in an increase in methylglyoxal concentration during the neutrophil studies, potentially explaining the increase in NET release; however, methylglyoxal has been found to inhibit MPO release, contradicting this theory [81]. Additionally, the electrospinning process could be catalyzing a chemical change in the Manuka honey during template fabrication that renders it less effective in reducing NET release. While glucose is a stable carbohydrate and unlikely to react with HFP, fructose is more reactive than glucose, and its interaction with HFP may be responsible for the cytotoxicity of electrospun templates with high concentrations of Manuka honey [82]. Hydroxymethylfurfural (HMF, or 5-hydroxymethylfurfural) is a cyclic aldehyde produced by the degradation hexoses such as fructose [73]. This reaction naturally occurs in Manuka honey as its high simple sugar content and low pH favor this reaction, causing an increase in a batch’s HMF content over time [83]. Additional exposure to acidic conditions and high temperatures can accelerate this reaction [73]. HFP has been demonstrated to extract HMF from reaction systems with fructose [84]. Manuka honey is approximately 33% fructose, providing a significant potential for HMF production in HFP. In low doses, it has antioxidant and anti-inflammatory effects, but safe levels of HMF remain inconclusive [73]. Excessive exposure to HMF has demonstrated mutagenic, genotoxic, and organotoxic effects including malignant enzyme inhibition and DNA damage in several in vitro, in vivo, and preclinical tests [85]. This potential change caused by HFP exposure would explain why Main et al.’s study, which only used Manuka honey solutions not exposed to any biomaterial fabrication processes, demonstrated a continuous reduction in NET formation as Manuka honey concentration increased, while Minden-Birkenmaier et al.’s TES templates and the NFES templates in this study demonstrated an increase in NET release. More studies are needed to evaluate the effects of biomaterial fabrication processing on Manuka honey and its ability to modulate neutrophil activation. Additionally, if processed whole Manuka honey does not demonstrate the desired immunomodulatory effects in biomaterials, the incorporation of Manuka honey components, such as methyl syringate, into biomaterials should be studied to determine the effectiveness of the individual components in reducing NET release.

## 5. Conclusions

There is a need for an off-the-shelf vascular graft with regulated inflammation and minimal risk of mechanical mismatch to improve the long-term patency of synthetic grafts. Bioresorbable NFES vascular templates have shown promise in mitigating mechanical mismatch but have yet to be evaluated as an anti-inflammatory drug delivery system. The Manuka honey NFES templates show promise in facilitating angiogenesis via transmural ingrowth of capillaries, as all templates possessed pores large enough for endothelial cells to infiltrate through the outer walls of the grafts via the initial response. The mechanical characterization revealed that the 0.1% and 1% Manuka honey concentrations outperformed pure HFP templates in several tests and achieved several native vascular target values. The 0.1% and 1% templates lacked a significant increase in NET release from the unstimulated control, suggesting that the addition of Manuka honey into the templates did prevent an increase in NET release. However, factors such as potential changes in honey composition during electrospinning and template architecture may have influenced the effectiveness of Manuka honey in modulating neutrophil activation as none of the Manuka honey-incorporated templates significantly reduced NET release compared to the pure HFP template.

Future research should focus on characterizing the impact of biomaterial fabrication processes on the effectiveness of Manuka honey in inhibiting NET release and exploring other specific Manuka honey components as biomaterial additives. Additionally, template fabrication should be optimized for mechanical strength and honey release, and the eluted honey’s ability to modulate NET release independent of templates should be assessed.

## Figures and Tables

**Table 1:** Fiber diameter, pore size, and wall thickness of the vascular templates. Raw data available in Supplementary Spreadsheets S1 and S2.

	Fiber Diameter (μm)	Pore Size (μm)	Wall Thickness (μm)
Pure HFP	3.44 ± 0.25	86.6 ± 21.2	207 ± 20
0.1% Manuka Honey	3.39 ± 0.52	74.5 ± 23.8	214 ± 8.6
1% Manuka Honey	3.38 ± 0.42	96.2 ± 19.7	236 ± 33
10% Manuka Honey	3.40 ± 0.33	128 ± 38.8	197 ± 15

## Data Availability

The original contributions presented in this study are included in the article/Supplementary Material. Further inquiries can be directed to the corresponding author.

## Supplementary Materials

The following supporting information can be downloaded at https://www.mdpi.com/article/10.3390/bioengineering12111270/s1, Supplementary Spreadsheet S1 (containing raw data for the pore sizes in Table 1), Supplementary Spreadsheet S2 (containing raw data for wall thickness inraw data in Table 1 and Figure 4, Figure 5, Figure 6, Figure 7 and Figure 8), Supplementary Spreadsheet S3 (containing raw data for Figure 9 and Figure 10), and Supplementary Spreadsheet S4 (containing raw data for Figure 11 and Figure 12).
